# Nutritional and Antioxidant Properties of *Moringa oleifera* Leaves in Functional Foods

**DOI:** 10.3390/foods11081107

**Published:** 2022-04-12

**Authors:** Rocío Peñalver, Lorena Martínez-Zamora, José Manuel Lorenzo, Gaspar Ros, Gema Nieto

**Affiliations:** 1 Department of Food Technology, Food Science and Nutrition, Faculty of Veterinary Sciences, Regional Campus of International Excellence “Campus Mare Nostrum”, 30071 Espinardo, Spain; rocio.penalver@um.es (R.P.); lorena.martinez23@um.es (L.M.-Z.); gros@um.es (G.R.); 2 Centro Tecnológico de la Carne de Galicia, Parque Tecnolóxico de Galicia, 32900 Ourense, Spain; jmlorenzo@ceteca.net; 3 Área de Tecnología de los Alimentos, Facultad de Ciencias de Ourense, Universidad de Vigo, 32004 Ourense, Spain

**Keywords:** moringa, mineral, health, nutrition, chemical composition, bioaccessibility

## Abstract

*Moringa oleifera* is a tree cultivated originally in northern India, whose ancient use as a medicine has demonstrated its antioxidant and anti-inflammatory properties. Due to its richness in minerals and macronutrients, the antioxidant capacity and the mineral bioaccesibility were assessed. In addition, the chemical composition, amino acid, fatty acid, and mineral content were also evaluated. The performed analysis reported a high content of proteins and low content of lipids in the chemical composition. Regarding the mineral content, Ca and Fe presented high bioaccessibility; K, S, Ca, and Fe being the most concentrated elements. The obtained values using FRAP, ABTS, and ORAC methods showed high antioxidant capacity, directly related to the increased content of phenolic compounds. In view of the results, *Moringa oleifera* can be incorporated into the diet as a functional ingredient or as a fortifier of any kind of food. The important source of minerals, phenolics, proteins, unsaturated fats, and folates make it an excellent extract with beneficial properties.

## 1. Introduction

Species and herbs have been used for centuries due to their medicinal properties. This is the case of the originally Indian tree *Moringa oleifera,* native of subtropical and tropical areas, but widely distributed in other regions of India, Asia, Africa, South Florida, Caribbean Islands, and South America [1,2,3,4]. Particularly, the pods and seeds of the tree *Moringa oleifera* are useful for water purification, due to its content in a cationic polyelectrolyte that has proven effective in treating water for human consumption (turbidity removal), causing colloidal particles to clump together and facilitating their removal by decantation or filtration. Furthermore, the chemical composition of the pods, seeds, and leaves of this tree makes them an important source of nutrients and micronutrients to be incorporated in our daily balanced diet.

Over the past several decades, agri-food industries developed their production chains to satisfy the food demands related to the new consumer requests and the growth of the population [5].

Consumers are looking to the promotion of the consumption of healthy foods, with increased interest in functional ingredients and nutraceutical foods, which can provide positive effects to the human body; specifically, since two years ago when the SARS-CoV-2 virus appeared and has remained in our lives [6].

The moringa tree is known by regional names such as Benzolive, Drumstick Tree, Horseradish, Mulangay, Marango, Sajna, Kelor, Saijihan, and Mlonge [7]. It is mainly used for human and animal food, which has various uses in agriculture, industry, and medicine [8,9,10]. All parts of the plant (pods, seeds, leaves, and roots) are consumed, as fresh and cooked, due to the fact that they are a complete food with a pleasant flavor [11,12,13].

The main contributions made by moringa in terms of macro and micronutrients are found in the leaves. This is the part of the moringa most used due to its high protein value. They are also rich in antioxidant components, among which the isothiocyanates stand out as one of the main carriers of anti-carcinogenic and antibiotic properties [14]. The contents of anti-nutritional components in its leaves such as tannins, lecithins, and protease inhibitors are insignificant; for this reason, they are edible in their totality. Moreover, they contain a balanced profile of essential amino acids and are an important source of vitamins A, C, and antioxidants [15]. Therefore, this plant has a nutritional profile to supply the needed micronutrients in a healthy diet, making us wonder whether it will be a useful alternative to fight against food spoilage [16].

Thus, the nutritional level of moringa has a great importance, since it has all the valuable amino acids, vitamins, and minerals even in greater amounts compared to other foods typically considered as such by their nutritional properties. In addition, the tree requires little agricultural care, grows quickly (up to 3–5 m in a year), and is resistant to drought. This last characteristic, together with the low cost of production, makes it ideal for cultivation in extensive desert or semi-desert areas of the African tropics, where there are serious problems of hunger, malnutrition, and undernourishment [17]. Recently, a high degree of interest was focused on the nutritional properties of moringa in occidental countries where it was not a native plant [18,19].

Moringa is proving to be a first class resource with low production costs to prevent malnutrition, anemia, and multiple pathologies such as child blindness associated with vitamin and element deficiencies in the diet [14]. In this sense, the objective of the study is to determine the chemical composition, nutritional value, and antioxidant activity of moringa leaves and their bioaccessibility to make a strong recommendation for their consumption in a balanced diet.

## 2. Material and Methods

### 2.1. Plant Material

Moringa powder obtained from 100% dried moringa leaves was supplied by KeyPharm Laboratories (Oostkamp, Belgium).

### 2.2. Extract Characterization

The moisture determination was assessed by weight difference in 3 g powder before and after drying for 24 h in a forced air oven at 110 °C [20]. The total organic matter analysis was performed based on the complete incineration of 1 g extract in a muffle oven at 525 °C for 24 h [20]. The Kjeldahl procedure was followed for the determination of the total protein content [21]. For that, 2 g of fresh sample were weighed, and 7 g of catalytic mixture and 15 mL of 96% sulfuric acid were added. The tubes were placed in a heating block, reaching 450 °C for 1 h. After cooling, a 4 min distillation was carried out with the addition of 32% NaOH until the neutralization of the mix. The product of the distillation had 0.1 N HCl. The next formula was used to calculate the total protein content (%), V being the total volume (mL) used to neutralize the mix, and W the weight of the sample.

(1) $$\mathrm{Raw}\;\mathrm{protein}\;\left(\%\right)=\frac{V\left(\mathrm{HCl}\right)\times 0.1\times 1.4\times 5.7}{W}$$

A Tecator Soxhlet extractor was used to determine the total fat content from 1 g of dried sample [21]. For that, the sample was kept into a cellulose cartridge and 50 mL ether were added. The ether heated to 80 °C extracts the fat from the sample and falls into the aluminum cups that were previously weighed. Finally, the fat content was calculated using the difference in the weight of the aluminum cups.

For the determination of the fiber, the procedure 985.29 of the AOAC [21] was followed. For that, 1 g of the moringa powder was weighed and 50 mL of 0.05 M phosphate buffer pH 6 were added. After this, the sample was placed in the bath at 95 °C for 30 min and α-amylase (40,000 U/mL) was added. After that, the pH was adjusted to 4.5, and the enzyme amyl-glucosidase (6 U/mg) added to again reach the temperature of 60 °C for 30 min. After finishing the digestion, ethanol was added to precipitate the fiber before filtering. At the end of the filtration, the residue was left in the oven and once dried, the total dietetic fiber (TDF) was determined applying the following formula:

(2) $$TDF\left(\%\right)=\frac{\frac{R1+R2}{2}-\left(P+A\right)}{\frac{W1+W2}{2}}\times 100$$

R1; R2: weight of the sample residue.

P; A: proteins and ashes from the residue.

*W*1; *W*2: sample weights.

### 2.3. Antioxidant Activity

Samples of 0.1 g of moringa powder were placed in tubes and 12 mL 80% methanol were added. The extraction was carried out by mixing for 1 h in darkness at 4 °C. The extracts were centrifuged at 3220× *g* for 10 min at 4 °C. The supernatant was collected, and a solution of 8 mg/mL was kept at −80 °C until analysis.

The antioxidant activity of the moringa extracts was evaluated following three different methods: Oxygen Radical Absorbance Capacity (ORAC), Ferric Reducing Antioxidant Power (FRAP), and the radical cation scavenging capacity against ABTS+ (2,2′-azino-bis(3-ethylbenzothiazoline-6-sulphonic acid)) radical.

Firstly, the hydrophilic antioxidant capacity measured using the ORAC method described by Ehlenfeldt and Prior [22] was carried out in a phosphate buffer (0.075 M, pH 7). For this, samples and Trolox standard solutions were pipetted into the wells of a 96-well black microplate, where 200 μL of 0.04 μM fluorescein were added. The microplate was kept for 15 min at 37 °C under darkness conditions and the reaction was started when 20 μL 40 mM AAPH (2,2′-azo-bis (2-amidine-propane)-dihydrochloride) were added. An excitation of 485 nm and an emission of 528 nm every 2 min at 37 °C for one hour and a half was set to measure fluorescence decay in the Bioteck Synergy HT (Winooski, VT, USA) microplate reader.

Secondly, the FRAP technique was performed by mixing FRAP reagent (20 mL 300 mmol/L acetate buffer pH 3.6 + 2 mL 20mmol/L FeCl_3_•6H_2_O + 2 mL 10 mmol/L TPTZ (2,4,6-tripiridil-s-triazina) in 40 mmol/L HCl) and the extracted sample [23]. The absorbance was read at 593 nm. All samples were analyzed in triplicate and the antioxidant activity was expressed as μmol of Trolox Equivalents (TE) per g.

Finally, the scavenging ability against ABTS+ radical was measured according to Re et al. [24]. For that, the absorbance of ABTS solution (7 mM ABTS+ with 2.45 mM potassium persulphate (1:1, v/v) pH 7.4) mixed with the methanolic extracted sample was measured at 734 nm. The chelating activity percent was calculated: ((Abs ABTS reagent—Abs sample)/Abs ABTS reagent) × 100.

### 2.4. Total Phenolic Content

The total phenolic content was determined using the Folin–Ciocalteu reagent and gallic acid as the standard following the method previously described by Singleton and Rossi [25]. For that, 0.4% Na_2_CO_3_ 2% NaOH were mixed with the extracted sample. After the addition of 1 N Folin–Ciocalteau reagent, the samples were incubated for 1 h under darkness conditions and the absorbance was read at 750 nm. The total phenolic content was shown as mg gallic acid equivalents (GAE) per g extract.

### 2.5. Determination of the Mineral Content

The mineral content was evaluated using inductively coupled plasma mass spectrometry (ICP-MS) (Thermo electron X7 inductively coupled plasma mass spectrometry, model X series, UK). The standards were diluted and used to calibrate the ICP-MS for mineral analysis in studied samples [26]. ICP-MS operating conditions were as following: nebulizer gas flow, 0.91 L/min; radio frequency (RF) 1200 W; lens voltage 1.6 V; cool gas 13.0 L/min; auxiliary gas 0.70 L/min.

### 2.6. Bioaccessibility Mineral

In order to assess mineral solubility and dualizability, a simulated gastrointestinal digestion of samples was performed using the method proposed by Minekus et al. [27]. For that, the simulated digestion fluids (salivary (SSF), gastric (SGF), and intestinal (SIF)) were prepared following the descriptions established by Minekus et al. [27] and the digestion process was divided into three phases: oral, gastric, and intestinal. Starting with the oral phase, moringa powder was mixed with SSF electrolyte stock solution (50:50; *w*/*v*) and pH was adjusted to 7. Human salivary α-amylase (75 U/mL) and 0.75 mM CaCl_2_ were added and an incubation at 37 °C for 2 min was performed. For the gastric phase, the previous mixture was mixed with SGF to obtain a final ratio of 50:50 (*v*/*v*) and the pH was reduced to 3.0 by using 1 M HCl. Porcine pepsin was added to achieve 2000 U/mL and samples were incubated at 37 °C for 2 h. Finally, for the intestinal phase, SIF was added to the mixture obtained (50:50) followed by CaCl_2_ to achieve 0.3 mM in the final digestion mix. pH was neutralized by using 1 M NaOH and porcine pancreatin and bile salts were added to 100 U/mL and 10 mM, respectively. This mix was incubated for 2 h at 37 °C. The supernatant obtained was filtered by 0.2 µm and the mineral content was measured as described above.

### 2.7. Determination of Folates

Folates were extracted from the sample following the procedure described by Knonings [28] and Pffeifer et al. [29]. For that, 1 g of sample was mixed to 25 mL of buffer (50 mmol/L HEPES + 0.2 g/L sodium ascorbate + 10 mmol/L 2-mercaptoethanol; pH 7.85) under a N_2_ atmosphere. The extracted samples were kept at 100 °C for 10 min, cooled, homogenized, and the pH was adjusted to 4.9. Enzymatic deconjugation and purification of samples were carried out according to Vahteristo et al. [30]. In total, 5 mL of the obtained mix were incubated for 3 h at 37 °C with 1 mL of hog kidney conjugase, as described by Gregory, Sartain, and Day [31]. Then, 1 mL of α-amylase preparation was used to inactivate the enzymes, kept at 100 °C for 5 min, cooled, and filtered (0.45 μm). This solution was purified in strong anion-exchange (SAX) cartridges connected to a Supelco 12-port vacuum manifold (conditioning: 3 mL of n-hexano, methanol, and MilliQ water (1:1:1) + 3 mL of purification buffer). After conditioning, the sample was loaded and eluted with 2 mL of elution buffer.

The separation and analysis of samples were performed with a HPLC/MS system consisting of an Agilent 1290 Infinity II Series HPLC as described by López-Nicolás et al. [32]. Folate standards used were: trihydrochloride tetrahydrofolic acid (H4), 5-Methyltetrahydrofolic Acid, 5-formyltetrahydrofolic acid, and folic acid supplied by Dr. Schirck’s Laboratory (Jona, Switzerland).

### 2.8. Statistical Analysis

All analyses were carried out in quadruplicates. A descriptive analysis was performed for the extract characterization and mineral and folate content. Regarding mineral bioaccessibility, the experimental design was a two-factor analysis (mineral compound and absorption phase) where an analysis of variance (ANOVA) was performed using SPSS software (vs. 27.0, IBM, Armonk, NY, USA). Statistical significance was set at *p* ≤ 0.05 and Tukey’s multiple range test was used to establish and separate means.

## 3. Results and Discussion

### 3.1. Chemical Composition of Moringa

The proximate composition of *Moringa oleifera* leaves is summarized in Table 1. As shown, the humidity of our moringa leaf powder was 7.23%, which is in close agreement with the data reported by previous authors, who established mean contents of water between 5 and 10% [33,34,35,36,37], although other studies have found lower moisture values than those obtained in our study [38,39,40,41,42,43,44,45]. Additionally, other studies have also found higher values of moisture content [46,47,48,49,50], which depends on the dryness of the original sample.

Regarding protein content, a quarter of *Moringa oleifera*’s weight presented as proteins (25.30% DW). That result agrees with data reported by Jongrungruangchok et al. [48], who noticed that the normal protein values are between 19.15% and 28.8%. Moreover, similar values were found by Oduro et al. [19], Olusanya et al. [33], Price [34], Rajput et al. [36], Okiki et al. [37], Mona [38], Yameogo et al. [39], Isitua et al. [40], Joshi and Mehta [42], Shiriki et al. [43], Offor et al. [50], Sánchez-Machado et al. [51], Fejér et al. [52], Dhakar et al. [53], and Fuglie et al. [54]. However, our value was higher than that obtained by Ogbe and Affiku [44], Amabye and Gebrehiwot [45], Umerah et al. [46], Fokwen et al. [49], and Valdez-Solana et al. [55]. While, on the contrary, our value was lower than that obtained by Castillo-López et al. [41], and Moyo et al. [47]. According to Castillo-López et al. [41], the protein content of *Moringa oleifera* changes due to weather variation, crop management, if they are cultivated or wild, the state of maturity of the plant at the time of collection, and the type of post-harvest processing. In this sense, we can appreciate the richness in proteins of this extract which, together with its high content in minerals, Fe, K, and Mg, can justify previous reports developed by cited authors, who exposed the ability of moringa to facilitate muscle recovery. These affirmations make the extract obtained from the dried leaves of this plant an excellent food complement to combat malnutrition in at-risk groups (elderly and children) and populations in developing countries.

In general, vegetables are not highly rich in lipids [46], so the fat content in *Moringa oleifera* is low (between 5 and 6%), which can vary between plants of the same family or species, due to external conditions such as humidity, nutrients, light, temperatures, or internal conditions such as age, which influence the development of physiological processes in plants [54,55,56]. Our value was similar to that reported by Oduro et al. [19], who found a content of 4.5%, and by Sánchez-Machado et al. [51] and Gopalakrishnan, Doriya, and Kumar, [57], who observed a 4.96% fat level. In contrast, studies developed by Mona [38], Yameogo et al. [39], Isitua et al. [40], Castillo-López et al. [41], Amabye and Gebrehiwot [45], Moyo et al. [47], Fokwen et al. [49], Fejér et al. [52], Verma and Nigam [58], and Witt [59] reported higher fat contents. As a matter of fact, a previous study indicated that *Moringa oleifera* leaves contained a high proportion of polyunsaturated fatty acids [40] and, hence, they are recommended for human consumption. Polyunsaturated fatty acids found in moringa leaves include omega-3 and -6, which have been demonstrated to help prevent and treat cardiovascular and neural diseases [60,61,62].

Regarding ash contents, the organic matter represents ~10% DW. This finding agrees with the data reported by Okiki et al. [37], Valdez-Solana et al. [55], and Oluduro [61]. Nevertheless, this value was lower than that obtained by Mutayoba et al. [62] and Sánchez-Machado et al. [51]. The ash content obtained in this study indicated that *Moringa oleifera* leaves are a good source of inorganic minerals [55]. Regarding carbohydrates, a high value was obtained, but this concentration was lower than that reported by Isitua et al. [40], Ogbe and Affiku [44], Amabye and Gebrehiwot [45], and Valdez-Solana et al. [55], who obtained values between approximately 54 and 63%. In this sense, these authors could account for the dietary fiber in the total amount of carbohydrates, which represented ~25% of the total weight and its addition would reach the values reported by them. Either way, the variation in the data obtained can be attributed to climatic factors, location, harvesting period, and nutrition of the plant [63,64,65]. These results justify the antidiabetic properties due to the hypoglycemic power of the fiber and the promotion of easy digestion.

### 3.2. Mineral Content and Its Bioaccessibility

The mineral content of the *Moringa oleifera* is shown in Table 2. In general, high levels of macrominerals were obtained, but among these, K (1752 mg/100g DW) was the most abundant element, followed by Ca (1475 mg/100g DW) and S (982,49 mg/100g DW), which was also shown by previous authors [34,37,46,53]. In this regard, Olusanya et al. [33] noticed that it could be used for the development of strong teeth and bones, which is a crucial need in children and pregnant women. Regarding the other macrominerals, high values were also obtained except for Na, that was the least abundant macromineral, but the data on Mg were lower than those found by other authors [34,35,36,37,43,51,53,61,62,63].

Furthermore, P content showed higher values compared to other studies carried out by Yameogo et al. [39], Dhakar et al. [53], and Thapa et al. [65]. In comparison, this plant contains much more P than a beef chop, so it could be an excellent source of P for bone and tooth formation [64,65,66].

After describing these values of the macrominerals mentioned above, it can be said that its consumption could cover the daily recommendations of Mg, Ca, P, and K noticed by AESAN [67].

**Figure 1:**
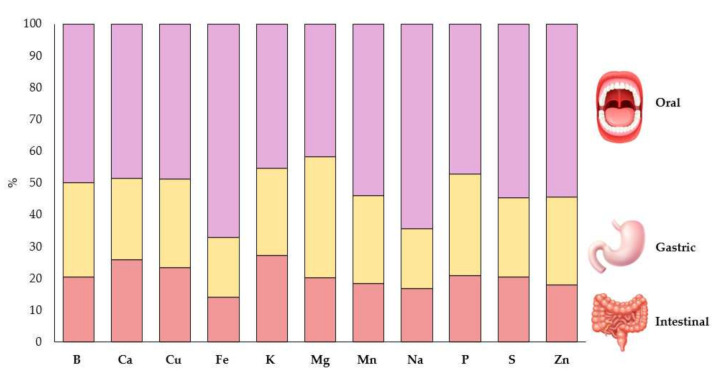
Bioaccessible minerals of moringa after the in vitro simulated gastrointestinal digestion (*n* = 4).

Regarding the microminerals, Fe, Mn, B, and Zn were the most abundant found in these leaves. In fact, our *Moringa oleifera* was found to have the highest content of Fe (25.14 mg/100 g DW). Our Fe value was higher than that obtained by Olusanya et al. [33], Umerah et al. [46], and Valdez-Solana et al. [55]. In this sense, the consumption of *Moringa oleifera* can be useful for providing the daily intake of Fe and to prevent anemia caused by Fe deficiency [68]. The Mn content obtained was higher than that found by Castillo-López et al. [41], Ogbe and Affiku [44], and Aslam et al. [69]. The next most important microminerals were B and Zn (3.54 and 2.04 mg/100 g DW, respectively), which bring health benefits such as protection for the immune system. For instance, B may be helpful in preventing osteoporosis [70], while Zn helps with the healing of wounds and regulates the enzymatic function [55,56,57]. Finally, Cu is found in smaller quantities, and higher values have been found in other studies, as once developed by Moyo et al. [47]. In this sense, because of its proximate composition and chemical content, *Moringa oleifera* has potential anti-inflammatory activities [71]. Our results showed that Moringa is also a source of Ca and Fe, both multifunctional nutrients essential for body metabolism [72]. In this way, in Figure 1, the mineral bioaccessibility of minerals studied is shown in all the phases of in vitro digestion.

As it can be appreciated, the mineral with the highest absorption in the oral phase was Na and the lowest was Zn, but in the gastric phase the highest was P and the lowest was Fe. Thus, the previous data regarding Fe absorption from the oral to the gastric phase had a high loss of bioaccessibility compared to Zn, which had the lowest percent of bioaccessibility in the oral phase, while in the gastric phase its loss of absorption was higher than Fe. In the intestinal phase, the mineral with the highest bioaccessibility was Ca and the lowest was Fe. Therefore, the Fe contained in *Moringa oleifera* suffers a loss of bioaccessibility in comparison with previous studies. For instance, a recent study reported that daily supplementation of 25 g of *Moringa oleifera* flour for 6 months reduced the prevalence of anemia in 1-year-old children [73]. In general, plant foods are rich in Fe but this Fe has a low bioaccessibility due to the fact that it is predominantly in the non-heme form and may be chemically complexed with a number of inhibiting factors, including phytate, polyphenols, or fibers [74]. Regarding Ca bioaccessibility, it displayed the opposite behavior in the intestinal phase: it increased in comparison with the gastric phase. This could be because the intestine interacts with digestive secretions and other components of the diet, forming complexes with stability and solubility constants dependent on intestinal pH, which facilitate the absorption [75]. In addition, some proteins and amino acids favor the bioaccessibility of this element [75].

### 3.3. Antioxidant Capacity

The methanolic extract of *Moringa oleifera* was evaluated for its antioxidant activity following several methods, which was directly related to the total phenolic content of moringa leaves. The results are summarized in Table 3.

The results showed that *Moringa oleifera* possesses an interesting antioxidant activity and acts as a good source of antioxidants due to the presence of several types of compounds such as ascorbic acid, flavonoids, phenolics, and carotenoids [76,77]. Antioxidant activity plays an important role in the protection against cell oxidation. In this sense, particularly, the antioxidant compounds found in Moringa leaves can be useful for the photoprotection against oxidative stress caused by UV exposure, which after prolonged exposure could lead to skin cancer.

The scavenging activity against the ABTS+ free radical indicates modest activity compared to other Indian plant extracts known for their interesting profiles of antioxidants, studied by Chanda et al. [78]. In addition, the value obtained is much higher when compared to the guava extract obtained by Thaipong et al. [79].

The FRAP method highlighted an interesting antioxidant profile, but the value obtained in this study was lower than that found by Baldisserotto et al. [80]. These data for moringa compared to other extracts known for their antioxidant capacity reported higher values, as, for example, Fernandes et al. [81], who showed comparable data from other plants known for their antioxidant activity such as *Rosmarinus officinalis* (361.57 ± 33.72 μmol Trolox/g).

Furthermore, the antioxidant activity shown in the ORAC method (against the oxygen reactive substances) indicated good antioxidant activity if compared with other plant extracts known for their high capacity, being higher than those obtained by Baldisserotto et al. [80] and by Castillo-López et al. [41].

As a matter of fact, as the phenolic compounds are one of the main compounds responsible for the antioxidant ability [79,80,81,82,83,84,85,86,87,88,89], these phytochemicals were also high in moringa leaves (Table 3), although these values were lower than those obtained by Castillo-López et al. [41], Baldisserotto et al. [80], and Özcan [89].

In this way, the high content of phenolic compounds, such as flavonoids and phenolic acids, is the main promotor of antibacterial, anti-inflammatory, and antitumor activity reported by cited authors. As a matter of fact, cell apoptosis derived from cancer development can be cured with moringa because of its antioxidant potential, which scavenges the reactive oxygen radicals, and, thus, avoids cell damage [79,80,81,82,83,84,85,86,87,88,89].

### 3.4. Folate Content

Among the vitamins found in *Moringa oleifera*, group B vitamins are the most abundant. In this sense, our results showed that there is a high level of folates in its leaves, where the most abundant folates were 5-Formyl-5,6,7,8-tetrahydrofolic acid (5FTHF), 5,6,7,8-tetrahydrofolic acid (THF), 5-methyl-5,6,7,8-tetrahydrofolic acid (5MTHF), and folic acid (FA) (Table 4), which agrees with Saini, Sivanesan, and Keum [90].

As shown, the total folate content is higher in comparison with other studies [89,90,91], such as Khalid Abbas, Elsharbasy, and Fadlelmula [91], although this may be due to the fact that in our study dried *Moringa oleifera* leaves were used, so they were more concentrated. Regarding these results, *Moringa oleifera* could cover part of the recommended daily intake according to the FESNAD [92], who advise a daily intake of between 300 and 400 µg/day. In this sense, this kind of product could be an interesting tool used to enrich foods focused on population groups at risk of deficiency in this type of nutrient, such as pregnant women.

Thus, the daily consumption of moringa leaves, incorporated in the preparation of main meals, may reduce the risk of degenerative and chronic diseases common in Western countries, where these diseases, resulting in part from poor dietary and exercise habits, are the leading cause of early death.

Furthermore, in developing countries, its regular consumption could help to alleviate the vitamin and mineral deficiencies that famine causes. Other advantages, such as its low cost and easy adaptation to certain climatic conditions, make its cultivation and harvesting a way to palliate the serious problem that world hunger is.

## 4. Conclusions

*Moringa oleifera* showed potential to be used as a functional ingredient for human food, shown by its protein, mineral, dietary fiber, and folate contents, and its low lipid content. It is also a source of Ca, Fe, Cu, and K, also having a high bioaccessibility of these minerals; therefore, it is a potential food enrichment source. In addition, it was demonstrated in this study that the concentration of phenolic compounds and antioxidant activity in *Moringa oleifera* leaves is enough to be considered as a potential source of antioxidant supplements. In general, it may have the potential to contribute to a better Fe content for breastfeeding mothers to combat the Fe deficiency problems and prevent many diseases including osteoporosis and cardiovascular disorders.

## Figures and Tables

**Table 1:** Proximate composition of the *Moringa oleifera* leaf (g/100g DW) *n* = 4.

Parameters	*Moringa oleifera* Mean Values
Moisture	7.23 ± 0.12
Protein	25.30 ± 0.27
Lipid	5.75 ± 0.21
Ash	9.95 ± 0.12
Carbohydrates	29.80 ± 4.55
Dietary fiber	24.97 ± 4.55

DW: dry weight.

**Table 2:** Mineral content of *Moringa oleifera* (mg/100 g DW) *n* = 4.

Minerals	*Moringa oleifera* Mean Values
Boron (B)	3.54 ± 0.01
Calcium (Ca) *	1.48 ± 0.01
Copper (Cu)	0.45 ± 0.05
Iron (Fe)	25.14 ± 1.13
Potassium (K) *	1.75 ± 0.02
Magnesium (Mg)	301.11 ± 2.08
Manganese (Mn)	7.21 ± 1.03
Sodium (Na)	133.11 ± 20.09
Phosphorus (P)	352.39 ± 1.19
Sulphur (S)	982.49 ± 11.56
Zinc (Zn)	2.04 ± 0.85

DW: dry weight. * values expressed in g/100 g DW.

**Table 3:** Antioxidant activity and total phenolic content of *Moringa oleifera* (*n* = 4).

Method	Mean ± sd
Total phenolic content (mg GAE/g)	32.90 ± 4.38
FRAP (μmol TE/g)	396.43 ± 17.12
ORAC (μmol TE/g)	3197.24 ± 19.65
ABTS (% scavenging activity)	41.40 ± 8.66

**Table 4:** Folate vitamers (µg/100 g DW) and total folate (expressed as µg folic acid equivalents/100 g DW) (*n* = 4).

Folate Vitamer	RT	m/z	Mean Value	Chemical Structure
THF C_19_H_23_N_7_O_6_	1.98	446.1783	22.40 ± 0.44	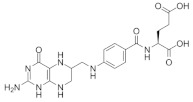
5FTHF C_20_H_23_N_7_O_7_	2.44	474.1732	16.98 ± 0.46	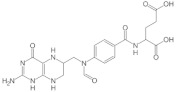
FA C_19_H_19_N_7_O_6_	3.35	442.147	16.57 ± 0.23	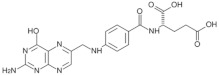
5MTHF C_20_H_25_N_7_O_6_	3.42	460.1939	13.82 ± 0.13	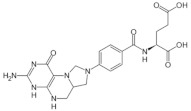
Total Folate content			69.77 ± 0.45	

THF: 5,6,7,8-tetrahydrofolic acid; 5FTHF: 5-Formyl-5,6,7,8-tetrahydrofolic acid; FA: folic acid; 5MTHF: 5-methyl-5,6,7,8-tetrahydrofolic acid.

## Data Availability

The data presented in this study are available within the article.
